# SWADAPT1: assessment of an electric wheelchair-driving robotic module in standardized circuits: a prospective, controlled repeated measure design pilot study

**DOI:** 10.1186/s12984-021-00923-2

**Published:** 2021-09-16

**Authors:** Emilie Leblong, Bastien Fraudet, Louise Devigne, Marie Babel, François Pasteau, Benoit Nicolas, Philippe Gallien

**Affiliations:** 1 Pôle MPR St Hélier, 54 Rue St Hélier, 35043 Rennes Cedex, France; 2 Living Lab ISAR, 54 Rue St Hélier, 35043 Rennes Cedex, France; 3 Institut National des Sciences Appliquées de Rennes-CNRS, Inria, Irisa-UMR6074, Rennes, France

**Keywords:** Smart wheelchair, Rehabilitation, Neurological diseases

## Abstract

**Objectives:**

The objective of this study is to highlight the effect of a robotic driver assistance module of powered wheelchair (PWC), using infrared sensors and accessorizing a commercial wheelchair) on the reduction of the number of collisions in standardized circuit in a population with neurological disorders by comparing driving performance with and without assistance.

**Methods:**

This is a prospective, single-center, controlled, repeated measure design, single-blind pilot study including patients with neurological disabilities who are usual drivers of electric wheelchairs. The main criterion for evaluating the device is the number of collisions with and without the assistance of a prototype anti-collision system on three circuits of increasing complexity. Travel times, cognitive load, driving performance, and user satisfaction are also analyzed.

**Results:**

23 Patients, 11 women and 12 men with a mean age of 48 years old completed the study. There was a statistically significant reduction in the number of collisions on the most complex circuit: 61% experienced collisions without assistance versus 39% with assistance (p = 0.038).

**Conclusion:**

This study concludes that the PWC driving assistance module is efficient in terms of safety without reducing the speed of movement in a population of people with disabilities who are habitual wheelchair drivers. The prospects are therefore to conduct tests on a target population with driving failure or difficulty who could benefit from this device so as to allow them to travel independently and safely.

## Introduction

Most people with disabilities need help getting around and often use technical mobility aids. These aids can have an assistance role, such as canes or walkers or, as in the case of the use of a wheelchair, a role of supplementing the action of the lower limbs when moving. For people with significant motor limitations, an electric wheelchair may be the only solution for long and safe journeys, providing them mobility and independence. Research has documented various benefits of using a wheelchair and has highlighted particularly improved mobility, better social participation, reduced caregiver burden, and reduced likelihood of orientation in the workplace [1].

The prevalence of people using a wheelchair tends to increase in developed countries, with an estimated range of 60 to 200 per 10,000 inhabitants [2]. However, while only 10% of wheelchair users use electric models [2,3], 25% of accidents are linked to their use [4]. In addition, 100,000 accidents involving wheelchair users were recorded in the United States in 2006, double the number of accidents recorded in 1991 [5]. The use of motorized devices for mobility assistance is not without risks and accidents can occur. In a more recent study on the prevalence of wheelchair accidents involving 95 participants, 54.7% of the subjects reported having had at least 1 accident in the past 3 years [6]. While falls and tips are the most common causes of accidents when using manual wheelchairs, collisions are the main problem for users of power wheelchair [7],^8^. Moreover Ummat noticed that 7.6% of the affected persons in wheelchair-related accidents were not the user but people hit by them [7]. Thus, due to cognitive disorders, behavioral disorders, or excessively disabling motor deficits (uncontrolled movements, non-functional spasticity, etc.), some of these people are in difficulty for driving a PWC due to the risk of putting themselves in a difficult situation or representing a danger to neighboring people. Indeed, if the risk of collisions with the environment seems too high, this may lead to restriction in using PWC and therefore to a limitation of mobility by extension of the daily autonomy of these people [9–11]. In these situations wheelchair skills training program, but also driving assistance may allow some patients to use an electric wheelchair [12]. Lack of technical assistance may increase activity limitations and restrictions on life in society and on social participation. This can impact the person’s life plan if the handicap cannot be compensated (Law of February 11, 2005, Article L.114). A study carried out by the Breizh Cerebral Palsy Network has shown the negative impact of limitations in moving around on the quality of life [13].

The importance of improving the quality of life, user autonomy, and social inclusion through the development of dedicated smart technologies for driving assistance has been underlined by Helal and Edwards [14], 15. PWC would improve people’s autonomy and help improve self-esteem [16] while having a positive impact on social participation. Indeed, Mortenson [17] showed the correlation between the daily distance traveled and participation in other activities for 246 dependent elderly people. In this context, a PWC driving assistance system could benefit people who are currently not eligible for the use of a PWC by improving safety when driving and thereby reducing wheelchair accident rates.

The problems of assistance to navigation in PWC have for several years been at the heart of the research themes of many research laboratories and have been addressed during several collaborative projects such as the NavChair [18], Radhar [19] Project Sysiass [20], and Coalas [21]. The semi-autonomous and autonomous PWC navigation assistance solutions developed within the framework of these projects use fragile and expensive sensors and are conventionally based on algorithms requiring computing power, reducing the battery and therefore the autonomy of the PWC. In addition, these solutions use bulky and fragile multi-sensor systems, resulting in a modification of the physical configuration of the PWC on which they are equipped. Finally, these solutions are not generic and cannot be adapted to all the different PWC models.

The main drawback of these systems remains the final cost of the solution and the lack of clinical trials leading to technology transfer for use by end-users [22–24].

In all the studies evaluating this type of system, safety is one of the main judgment criteria. Evaluation can be made directly by counting the number of collisions with the environment or indirectly through the measurement of success in different driving tasks, defined specifically for the needs of studies or through standardized driving scores such as WST or PIDA [1, 25].

As part of the European ADAPT project (Assistive Devices for empowering disAbled People through robotic Technologies) [26], a driver assistance module has been developed to provide a solution allowing PWC users to travel safely.

The objective of this study is to highlight the effects of this robotic driving assistance module for PWC on the reduction of the number of collisions in standardized circuits in a population suffering from neurological disorders and moving in an electric wheelchair by comparing performance with and without assistance in order to validate safety, user satisfaction, and efficiency.

## Materials and methods

### Participants

For ethical reasons, participants in this first study were regular wheelchair users without any difficulty who are not a priori the targets for a power wheelchair driving assistance system. The objective was to validate the assistance system before proposing it to users in difficulty. We had the assurance for these expert patients that in case of failure of the assistance system they would not lose control of their wheelchair and that the safety would therefore be maximum.

#### Inclusion criteria

The inclusion criteria were:

- Being over 18 years of age,
- Having benefited from a prescription for an electric wheelchair and traveling with one for more than 3 months,
- Having the electric wheelchair as one’s main mode of travel,
- Having compatible physical measurements (weight, height) with the chosen chair (Quickie SALSA M2) as part of the development of the robotic assistance module,
- Having freely consented to participate in the study.

#### Exclusion criteria

Comprehension disorders that make it impossible to consent to the use of clinical results for research purposes were an exclusion criterion, as were motor disorders of the upper limb that require additional technical driving assistance (chin control for example). Likewise, patients who expressed difficulties impacting their indoor and/or outdoor driving safety and vulnerable people were excluded (hemiplegia with hemianopsia, severe ataxia of the upper limb for example).

### Procedure (Fig. 1)

**Fig. 1:**
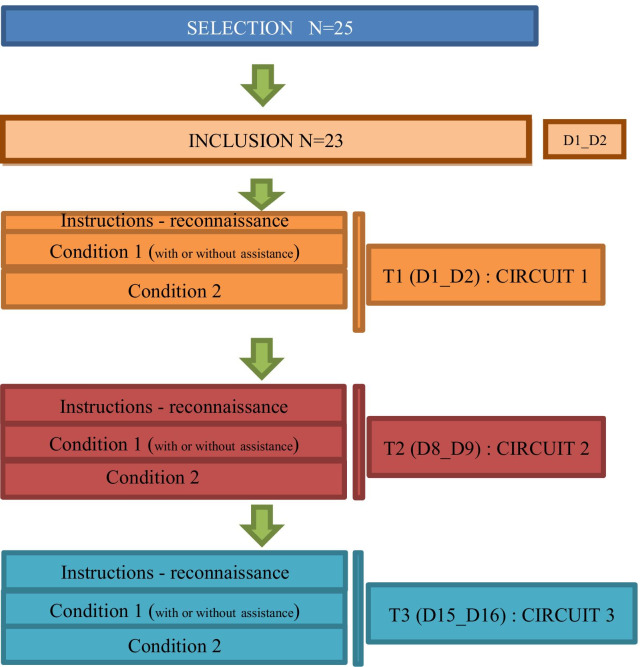
Design of study SWADAPT1

This is a prospective, single-center, controlled, repeated measure design, single-blind pilot study carried out at Pole MPR St Helier, Rennes, France in June 2019, having received the agreement of the CPP Nord Ouest I on May 27th, 2019.

During medical consultations, the investigating doctor presented the objectives and procedures for participating in the protocol in verbal and written forms. For patients who volunteered after a 10-day cooling-off period, the consent form was signed.

Circuits (D1 ± 1 – D8 ± 1 – D15 ± 1).

**Fig. 2:**
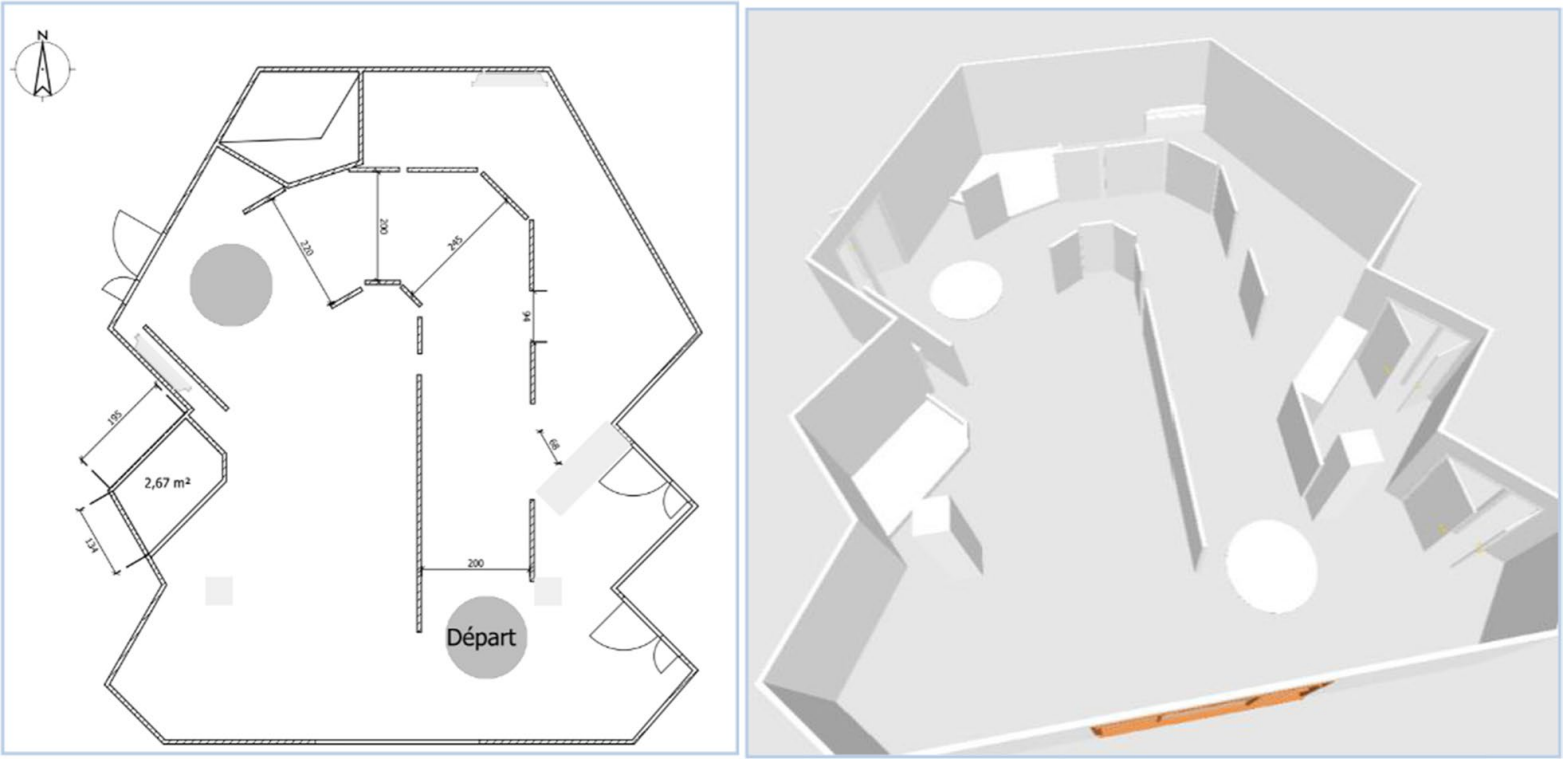
Plan and 3D model of circuit 1

**Fig. 3:**
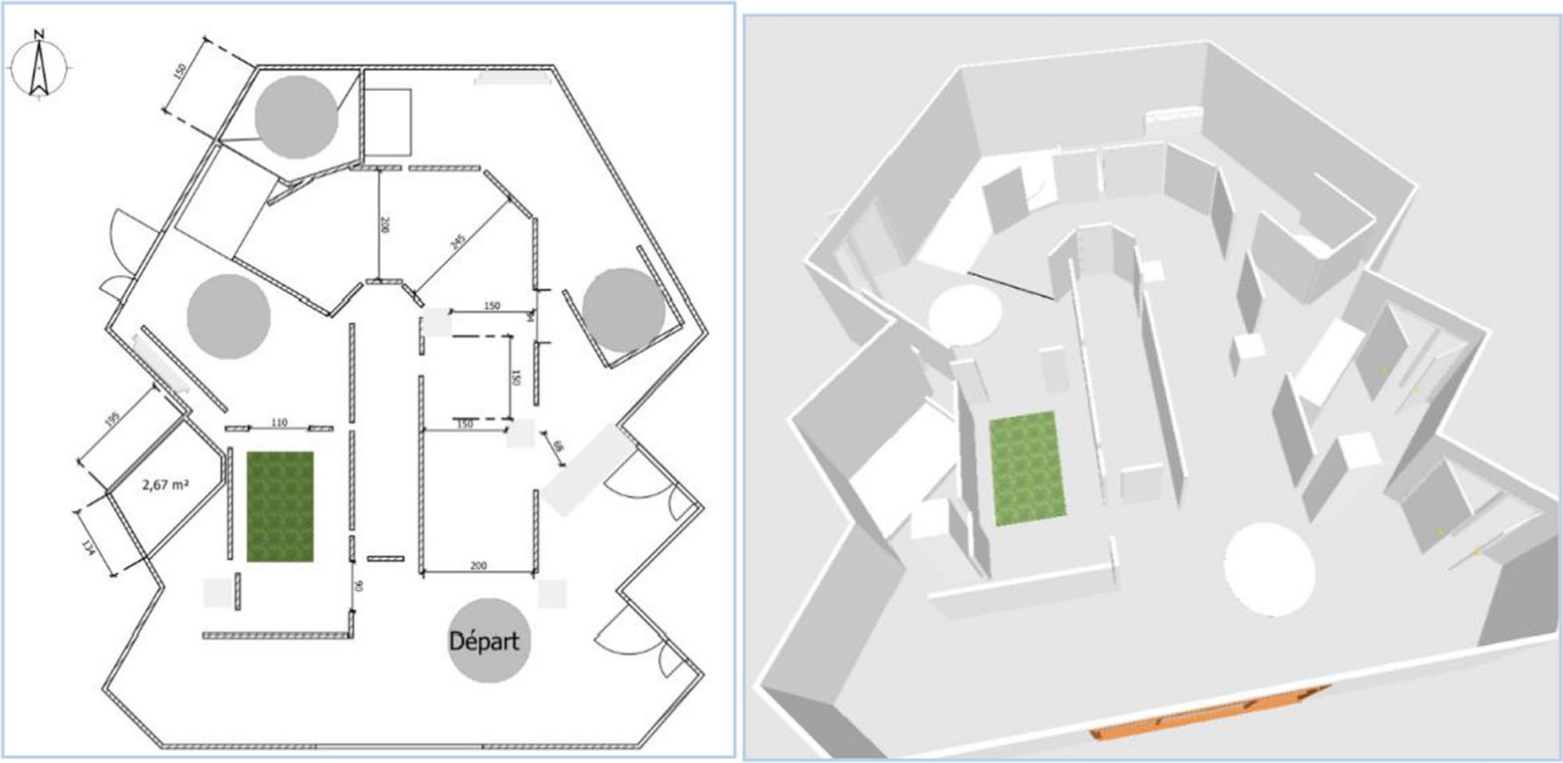
Plan and 3D model of circuit 2

**Fig. 4:**
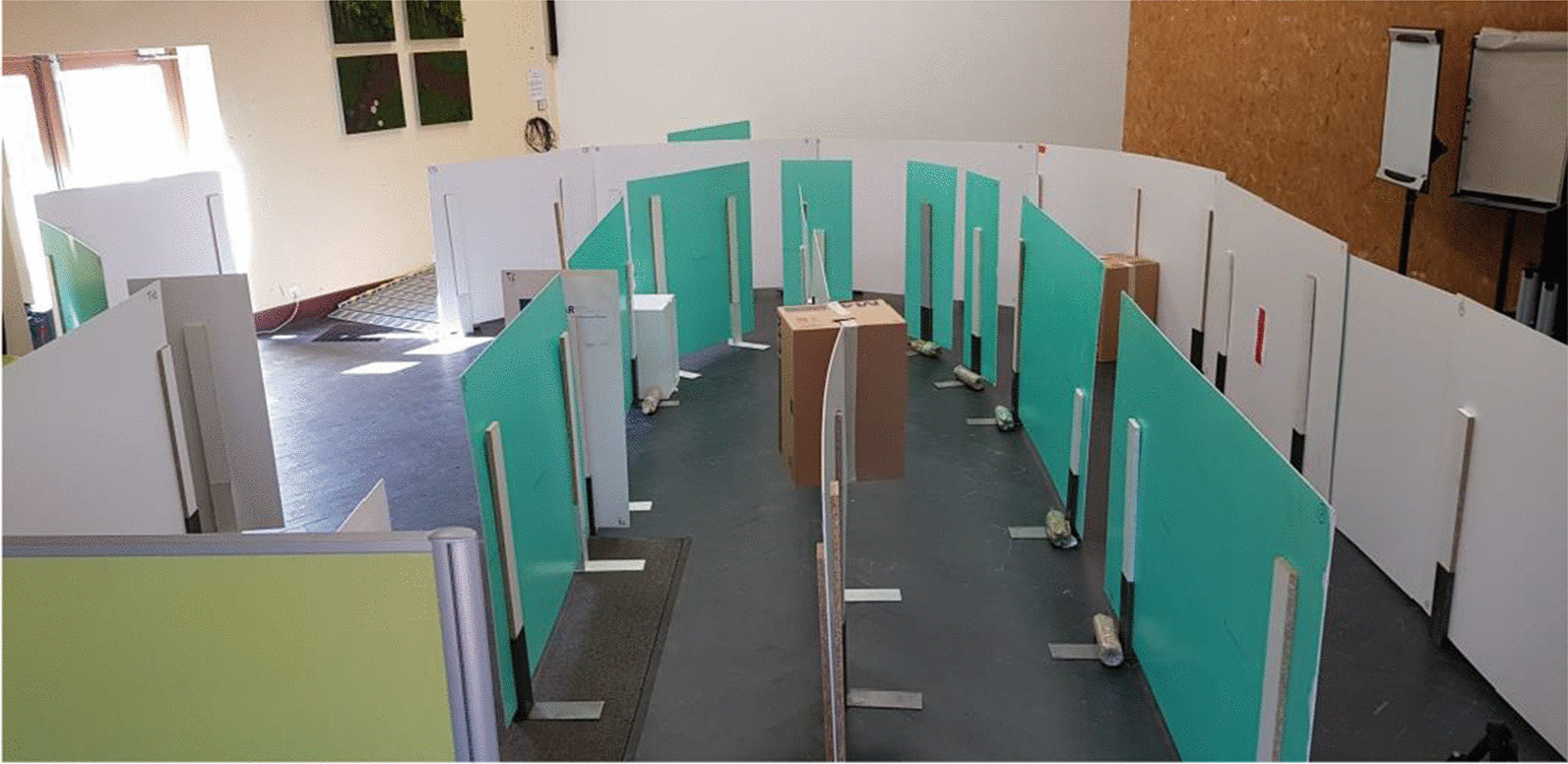
Detail of circuit 2

**Fig. 5:**
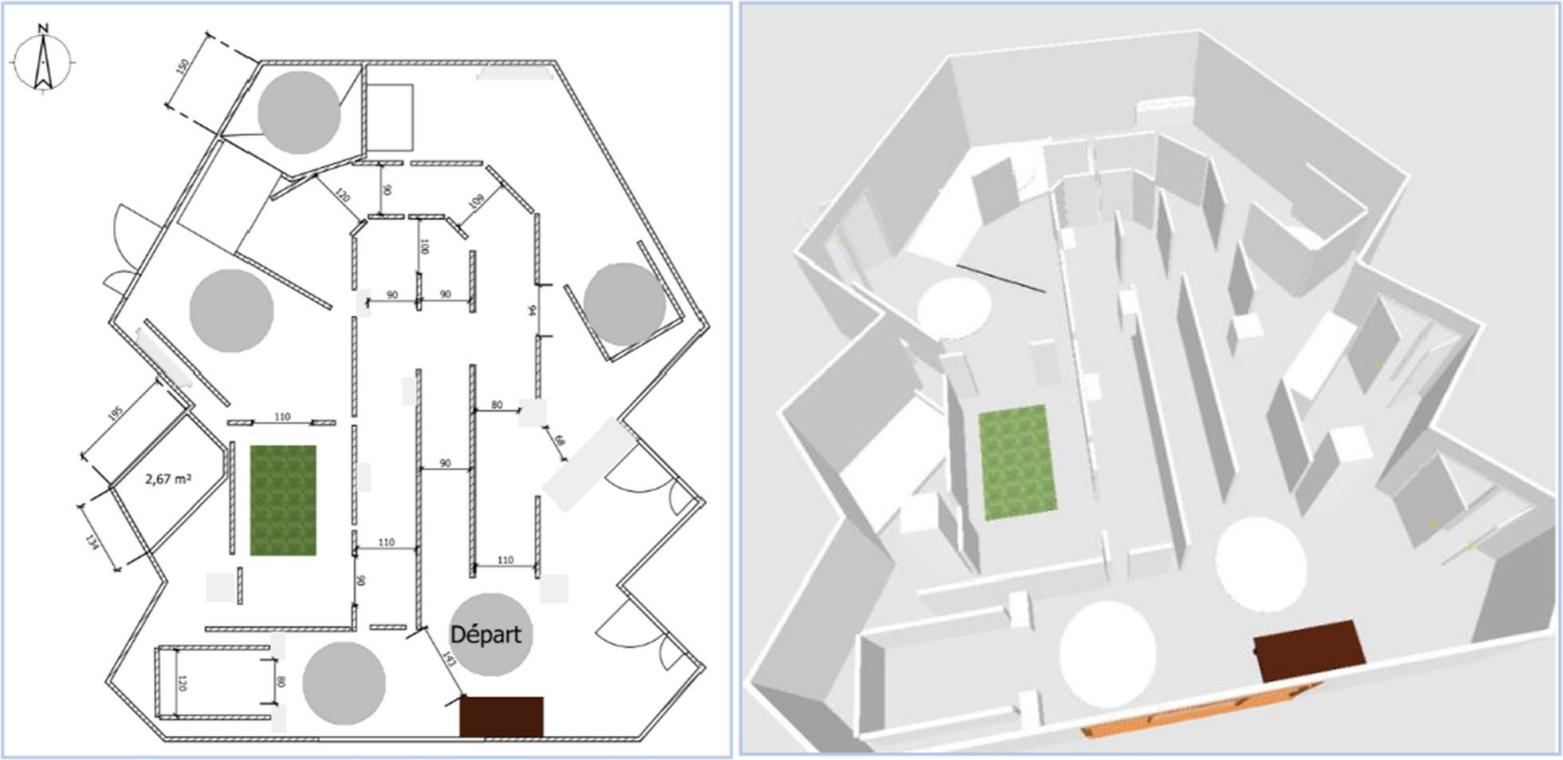
Plan and 3D model of circuit 3

As part of this investigation, the patients were followed for 15 days, at a rate of 3 visits to the Pole Saint-Helier:

- Circuit 1 (D1), Fig. 2,
- Circuit 2 (D8), Figs. 3 and 4,
- Circuit 3 (D15), Fig. 5.

The ADAPT project teams have defined three circuits intended to evaluate this first robotic assistance module. The circuits have been developed in collaboration with professionals in the training and evaluation of electric wheelchairs, drawing on data from the literature concerning the circuits and recommendations for evaluation [1,25,27–29].

The purpose of these circuits is to assess the benefits of the various assistance modules designed as part of the ADAPT project.

These circuits are of increasing difficulty, allowing assistance to be tested through a sequence of tasks (Table 1), in a situation of reproducibility that a circuit can offer.

**Table 1:** The different tasks included in each circuit

Circuit	Task description
1	Wide corridor (2,5 m)
	Forward
	Wide turn
	U-turn on site
	Reverse
2	Fixed obstacle on the ground to be circumvented
	Low slope (5°) to go down and up
	Wide corridor / wide doorway
	Fixed obstacle on the ground to cross
	Low and moderate slope (5° and 10°) to go down and up
3	Fixed obstacle in height
	Emergency stop
	Stop with precision
	Walk along a wall
	Narrow corridor (1.5 m)
	Table: setting up
	Double stain
	Elevator simulation: forward entry and reverse exit
	Restricted space
	Moving obstacle to bypass

These three circuits have been created to meet specific electric wheelchair driving scenarios, corresponding to low to moderate sedan driving requirements [27, 28].

The circuit is made up of flexible, mobile, and removable plastic walls that promote safe circulation: collision causes the structure to move.

The slopes use rails specifically dedicated to PWC traffic, secured by guardrails and lined with wheel guards. The dimensions of width paths are 2 m for C1, 0.9 to 2 m for C2 and 0.75 to 1.10 m for C3.

The first circuit (Fig. 2) makes it possible to test elementary driving tasks; the second circuit (Figs. 3 and 4) adds tasks of moderate complexity; the third circuit (Fig. 5) includes tasks of more marked difficulty. The different tasks are described in Table 1. The tasks included were chosen based on the one hand on a survey of 350 professionals competent in PWC driving training, carried out as part of the ADAPT project, and on the other hand on data from the literature. The different measurements were carried out by 2 independent trained occupational therapists in double blind.

To be able to assess the benefit of the assistance module on a PWC, the different circuits are carried out under the 2 following distinct conditions in a randomized order, known only to the engineer who programmed the assistance module and who was blinded to the evaluators and the patients.

- PWC with activated assistance module,
- PWC with deactivated assistance module.

Each patient therefore performed 6 trails per circuit spread over 3 days (see Fig. 1). Before running these courses, they disposed of 5 min to get familiar with the PWC with the assistance module.

### Device

As part of this investigation, a standard QUICKIE Salsa M^2^ PWC, width of 62 cm from Sunrise Medical (Fig. 5—CE marked Class I medical device) was made available to patients for the various courses.

The sensors are integrated into the structure of the standard PWC and they do not modify the structure and the overall shape of the PWC in any way. Frequency of control signal was the same for all participants: 50 Hz. These are Time-of-Flight infrared sensors distributed to the front right, front left, rear right, and rear left of the wheelchair [30, 31].

The developed robotic assistance module is a semi-autonomous PWC driving assistance system, i.e. a control system shared with the user.

Intended to accessorize series, this system is made up of measurement modules (made up of ultrasonic sensors) and an on-board calculation unit connected to the sensors and to the wheelchair power module. The module’s sensors are placed in different places on the PWC:

- under the PWC footrests,
- behind the seat of the PWC,
- on the lateral sides of the PWC.

**Fig. 6:**
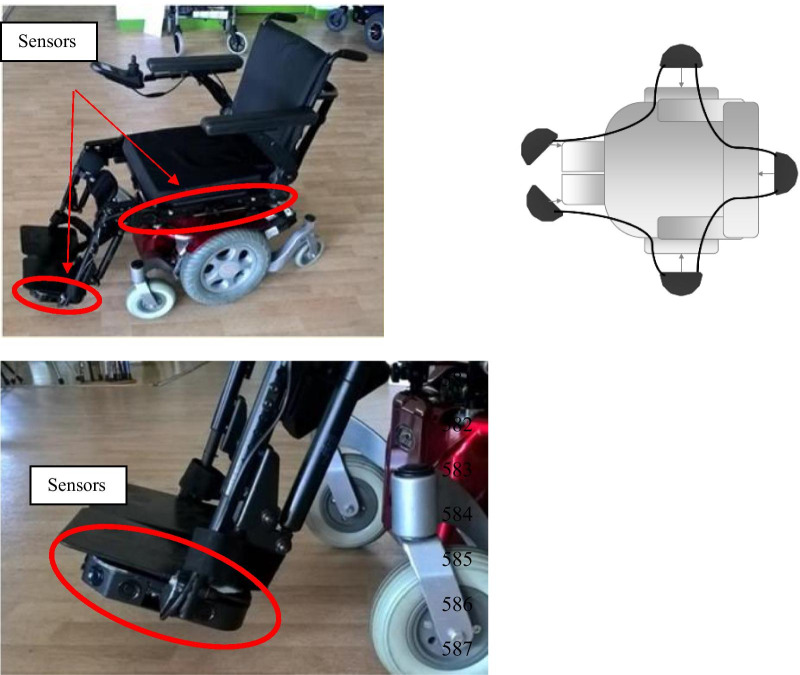
The smart wheelchair with US sensors

Thus, the module is not in direct contact with the user (see Fig. 6).

Constraints on the speed of the PWC are calculated from the distances measured around the chair. Deduced from the environment, these constraints are subsequently merged with the user’s command (data from the control unit) to recalculate the speed to be applied to the wheelchair to gradually modify its trajectory and to avoid collisions with the environment, while respecting the user’s intention as much as possible (Fig. 2).

With the module activated, the PWC will stop on a frontal arrival in front of a positive physical obstacle (protruding from the ground, unlike a negative obstacle such as a hole or a curb ledge) at about fifteen centimeters. In the event of an angled arrival, the PWC will autonomously circumvent the obstacle by skirting it. At any time, the user can interrupt the assisted maneuver by releasing the control, as with the traditional PWC.

The chair control module is based on the use of a license from chair electronics manufacturer Penny & Giles, obtained by INSA Rennes. The system fully reuses the safeguards provided by Penny & Giles. Thus, when the assistance is activated, the safety installed on conventional PWC consisting of stopping the wheelchair when the joystick is released is maintained. The PWC therefore stops as soon as the joystick is released.

### Objectives

The main objective is to highlight the effect of a robotic driving assistance module for PWC on the reduction of the number of collisions in standardized circuits in a population suffering from neurological disorders and moving in an electric wheelchair by comparing performance with and without assistance.

The secondary objectives were:

- To highlight the benefit of a robotic driving assistance module on driving speed,
- To highlight the value of a robotic driving assistance module on driving performance while carrying out defined tasks,
- To measure the impact of the module on cognitive load,
- To assess user satisfaction.

### Efficacy endpoints

The main endpoint is the number of collisions on different standardized circuits with and without activation of the robotic assistance module as evaluated by two independent occupational therapist evaluators. A collision is defined as a contact with real walls of the circuits. The average value over three passages for each circuit and each patient is considered. An agreement between the two evaluators is necessary to validate a collision.

The secondary endpoints are:

- the speed measured by the time to complete the course with and without the activation of the assistance system on various circuits in seconds,
- driving performance measured by the Wheelchair Skill Test items corresponding to the different routes, with and without the activation of the assistance system (WST, [1])
- the cognitive load of the tests under the 2 conditions as measured by the NASA-Task Load Index (NASA TLX, [32]). Indeed an important question is the cognitive load: does the use of a driving assistance increase the cognitive load? If so, this could be a difficulty for patients who have difficulty driving a wheelchair.
- satisfaction with the use of PWC under the conditions as assessed by the Ease of Use Questionnaire (USE, [33]).

Assessments were performed before and at the end of each trials.

### Statistical analysis

Statistical data were analyzed using the R studio software version 4.1.0, package rstatix v.0.7.0 (2021) on paired data N = 23. Quantitative descriptive data consisted of mean and standard deviation, median, and quartiles.

We used the paired samples Wilcoxon test to compare paired data as the samples were small with a 0.05 significance threshold. Furthermore, due to the small sample size, the size effect was calculated with r of Wilcoxon [34]. There was no missing data.

## Results

### Population

25 Users were contacted, all accepted to enter the study. Finally, 23 completed the study. The 2 patients left the study for personal reasons (a car breakdown and a broken leg following a fall at home independent of the study). The final sample consisted of 11 women and 12 men, with a mean age of 48 years (SD 11 years). Neurological pathologies were varied: 7 spinal cord injuries with a neurological level between C7 and L1, 5 multiple sclerosis with an EDSS score upper than 6, 5 cerebral palsies with GMF score of 3 and 4, 3 peripheral neuropathies with sensori-motor tetraparesis, 2 strokes, a left hemiplegia with hemineglect and a bilateral motor deficit after multiple brain lesion with restriction of the visual field, and 1 neuromuscular disease with tetraplegia. No patient had cognitive disorders.

### Number of collisions (Table 2)

**Table 2:** Number of collisions during the three circuits

Sers	C1 without assistance	C1 with assistance	C2 without assistance	C2 with assistance	C3 without assistance	C3 with assistance
N°1	0	1	0	0	0	0
N°2	2	0	0	0	3	0
N°3	0	0	0	0	0	2
N°4	2	0	0	0	2	0
N°5	3	1	0	0	6	0
N°6	0	0	0	0	0	1
N°7	0	0	0	0	0	0
N°8	0	0	0	0	1	0
N°10	0	1	0	0	2	1
N°11	0	0	0	0	1	0
N°12	0	0	0	0	0	2
N°13	3	0	0	0	1	3
N°14	0	0	0	0	0	1
N°15	2	2	0	0	0	0
N°16	2	1	0	0	0	0
N°17	0	0	0	0	1	1
N°18	1	0	0	0	2	2
N°19	0	1	0	0	4	0
N°21	0	0	0	0	4	0
N°22	0	0	0	0	6	0
N°23	0	0	1	0	8	2
N°24	0	0	0	0	5	0
N°25	0	0	0	0	0	0

Circuit 1: 31% of users without assistance experienced collisions vs 26%. Mean number of collision was 0.65 ± 1 without assistance and 0.3 ± 0.6 with assistance (NS).

Circuit 2: 4% of users without assistance experienced collisions vs 0%. Mean number of collision was 0.04 ± 0.6 without assistance and 0 ± 0 with assistance (NS).

Circuit 3: 61% of users without assistance experienced collisions vs 39%. Mean number of collision was 2 ± 2.4, median 1, without assistance and 0.65 ± 0.9, median 0, with assistance (p = 0.028) with a moerate effect size (r = 0.39) [35].

We can therefore conclude that the assistance had a significant effect on the score by reducing the number of collisions.

### Time to completion

The mean time on course 1 was higher in the “with assistance” condition than in the “without assistance” condition (87.04 s ± 6.59 versus 85.08 s ± 3.91). This difference was significant (p = 0.024), with a moderate effect size (r = 0.43) No difference was noticed for the circuit 2 (102.04 s ± 6.79 versus 102.09 s ± 6.92).

The mean time score on circuit 3 was significantly higher in the “with assistance” condition than in the “without assistance” condition (p = 0.018), with a moderate effect size (r = 0.50) (178.78 ± 16.71 versus 173.52 ± 14.62).

We can therefore conclude that the assistance had a significant effect on the time to completion. The course time increased when the assistance was activated.

### Driving performance (WST)

There was no significant difference between the conditions in terms of the overall driving ability scores for either circuit (p = 1, p = 0.35, and p = 0.10, respectively).

### Cognitive load (NASA-TLX)

There was no significant difference between the conditions in terms of the overall cognitive load scores for either circuit (p = 0.25, p = 0.67, and p = 0.31, respectively).

### User satisfaction (USE) (Table 3)

**Table 3:** Score of USE

		Usability	Ease of use	Ease of learning	Satisfaction
		Mean scoreS	Mean scoreS	Mean scores	Mean scores
Circuit 1	Without assistance	4.78 ± 2.4	6.27 ± 1.23	6.76 ± 0.66	5.67 ± 1.96
	With assistance	4.73 ± 2.2	6.17 ± 1.3	6.77 ± 0.55	5.44 ± 2.09
Circuit 2	Without assistance	5.26 ± 1.9	6.35 ± 1.07	6.67 ± 0.6	5.8 ± 1.7
	With assistance	5.03 ± 2	6.27 ± 1.3	6.5 ± 1.13	5.77 ± 1.75
Circuit 3	Without assistance	5.38 ± 1.85	6.03 ± 1.6	6.57 ± 0.9	5.59 ± 1.87
	With assistance	5.26 ± 1.83	6.13 ± 1.3	6.58 ± 0.88	5.61 ± 1.8

There was no significant difference between the conditions in terms of the U.S.E scores or sub-scores for either circuit (p = 0.69 p = 0.92, and p = 0.80, respectively).

## Discussion

Our study reports statistically significant results on the number of collisions, leading to the conclusion that the electric wheelchair driver assistance module is effective in enhancing user safety, despite a difference in travel time. Besides the efficiency of the system, it is important to emphasize the safety aspect. The assistance system appeared to be reliable. No technical problem was observed during the tests.

As this is a pilot study on a prototype power wheelchair add-on, several factors for SWADAPT1 could not be calculated. Nevertheless, the results relate to a population of 23 participants, which is relatively high in view of the data existing in the literature. The sample was heterogenous in terms of diagnosis, but each patient was his own control in the study so the pathology cannot influence the statistical final results. As part of the European ADAPT project, the driver assistance module makes it possible to navigate in safety as shown by the significant reduction in the number of collisions thanks to a system adaptable to any model of electric wheelchair based on inexpensive ultrasound technology.

### Semi-autonomous solutions including existing anti-collision assistance

Previous studies have been devoted to the evaluation of anti-collision systems and have underlined the interest of such system [23,24,36–39]. The assistance systems vary from one study to another as does the evaluation methods. Several modalities have been experimented: visual, haptic, sound feedback. McGarry tested an anticollision system coupled with a ground line tracking solution reducing the field of exploration for users. On his side Simpson studied the benefit of a voice-controlled collision avoidance solution. The system experimented by Boucher performed complex tasks autonomously (e.g. parking) to the PWC using a voice command.

Samples were often small and do not always involved users unlike our study. Thus Sharma et al. evaluated an anti-collision assistance system for PWC in 19 healthy people blinded by a blindfold on a standardized circuit [37, 24]. Boucher compared the driving performance of 17 participants, 8 of them were healthy participants. Studies involving only users involve much smaller numbers: 4 children suffering from cerebral palsy for McGarry et al., 7 people with visual disturbances for Sharma and 5 elderly people with moderate cognitive impairment for How. Concerning the methods of assessment, collisions remain the main criteria during a driving test on standardized circuit. But several other tests were also used by the different teams. The driving performance of the participants in Boucher study was assessed using a version of the standardized Wheelchair Skills Test adapted for robotic PWC, with a reduction of more than 60% in the number of collisions when the navigation assistance module was activated [24].

An increase of mental load due to the assistance system may be a difficulty for patients suffering from cognitive disorders who are one target of the assistance module. Sharma and How assessed the mental load with the NASA-TLX, no increase of the mental load was also found [37,40]. How also used the psychological impact through the PIADS (Psychosocial Impact of Assistive Devices Scale), and noticed good acceptance for all people assessed as in our study with the USE.

### Tests on circuits

One criticism that can be made is related to the evaluation of driving on a standardized course during this first phase and not in a daily life situation. However, the on-road test of a PWC allows a precise assessment of the technical performance and/or driving capabilities of the PWC. Indeed, the use of a course for evaluating the use of a PWC has the advantage of reproducing strictly identical situations and thus of working on the precision of the trajectory, particularly on the avoidance of obstacles, on a persistent difficulty such as going through a door or on learning by repeating a course. This exercise also makes it possible to compare the capacities of users [12] or, in the same way, to evaluate a PWC and/or a device adjoining this wheelchair [20,41]. In addition, by offering the user the opportunity to get into specific situations in complete safety (avoidance of cardboard obstacles, delicate maneuver in a secure environment, etc.), the course is of definite interest concerning the learning and validation of basic driving skills of a PWC.

Among the studies regarding a tool for learning and/or evaluating the capacities for using PWCs, the method and the quality of performance of each task is observed. The travel strategy [24,42,43], the completion time [37, 39, 44], and the number of collisions [39] are notably evaluated.

Among the studies that evaluate an “intelligent” PWC driving assistance device, priority is given to the evaluation of the system, i.e. of the performance of the wheelchair equipped with the system in the face of an obstacle, particularly on the ability to move around and maneuver [43, 24] by comparing the number of collisions and the time to complete the course with and without assistance [37, 39]. The practical evaluation of an on-course PWC is regularly accompanied by an evaluation questionnaire such as the QUEST [45], the WST-Q [46], and the NASA-TLX [32].

Due to a lack of precise information in studies including course evaluations, it is difficult to reproduce the courses and scenarios presented in the literature, which has the consequence of slowing down the possibility of cross-referencing the results of studies using similar routes. Among these studies, several tools have nevertheless proved their validity and reliability: the WST-P [1], the PMCDA [29], and the PIDA [25].

### Strengths and weaknesses

This is one of the few studies to validate a driver assistance system on a sample larger than 20 with an adequate methodology.

The sample size of our study remains small but it is composed of patients experienced in driving power wheelchairs. The first two circuits seem too simple for expert drivers and therefore not very discriminating. Finally, the third circuit proved to be the most suitable for determining the effectiveness of the assistance system with a fairly high effect size.

Moreover this study concerns expert patients who do not need this technological solution. A validation of the clinical interest will be necessary in a second study with subjects in difficulty for power wheelchair driving.

## Conclusion

This article presents a study to evaluate the use of an electric wheelchair driver assistance system. The objective of the solution developed within the framework of the ADAPT project is to improve safety conditions when driving a PWC, to make it possible to reduce the rate of wheelchair accidents, and to facilitate access to PWC for people who are not currently eligible. The study assessed the efficiency of such a PWC driver assistance system on the driving performance of regular drivers with disabilities to ensure safety. The reduction in the number of collisions confirms the interest of such a module among its target population. This does not modify the cognitive load of driving and meets the objectives of patient satisfaction. The next step is of course the conduct of a similar study to evaluate such an assistance module in prospective users who are currently not eligible, either due to driving difficulties or to not having access to such devices for safety reasons.

The authors report no conflicts of interest regarding the methods or the interpretation of the results.

## Data Availability

The dataset uses and analysis during the current study are available from the corresponding author on reasonable request.
